# Does Fear Increase Search Effort in More Numerate People? An Experimental Study Investigating Information Acquisition in a Decision From Experience Task

**DOI:** 10.3389/fpsyg.2018.01203

**Published:** 2018-08-03

**Authors:** Jakub Traczyk, Dominik Lenda, Jakub Serek, Kamil Fulawka, Pawel Tomczak, Karol Strizyk, Anna Polec, Piotr Zjawiony, Agata Sobkow

**Affiliations:** Wrocław Faculty of Psychology, SWPS University of Social Sciences and Humanities, Wrocław, Poland

**Keywords:** numeracy, decision from experience, fear, incidental affect, integral affect, search effort, uncertainty, emotion

## Abstract

The aim of this study was to investigate the effect of numeracy and the emotion of fear on the decision-making process. While previous research demonstrated that these factors are independently related to search effort, search policy and choice in a decision from experience task, less is known about how their interaction contributes to processing information under uncertainty. We attempted to address this problem and to fill this gap. In the present study, we hypothesized that more numerate people would sample more information about a decision problem and that the effect of fear would depend on the source of this emotion: whether it is integral (i.e., relevant) or incidental (i.e., irrelevant) to a decision problem. Additionally, we tested how these factors predict choices. We addressed these hypotheses in a series of two experiments. In each experiment, we used a sampling paradigm to measure search effort, search policy and choice in nine binary problems included in a decision from experience task. In Experiment 1, before the sampling task we elicited incidental fear by asking participants to recall fearful events from their life. In Experiment 2, integral fear was elicited by asking participants to make choices concerning medical treatment. Decision problems and their payoff distributions were the same in the two experiments and across each condition. In both experiments, we assessed objective statistical numeracy and controlled for a change in the current emotional state. We found that more numerate people sampled more information about a decision problem and switched less frequently between alternatives. Incidental fear marginally predicted search effort. Integral fear led to larger sample sizes, but only among more numerate people. Neither numeracy nor fear were related to the number of choices that maximized expected values. However, across two experiments sample sizes predicted the number of choices that maximized experienced mean returns. The findings suggest that people with higher numeracy may be more sensitive to integral emotions; this may result in more effortful sampling of relevant information leading to choices maximizing experienced returns.

## Introduction

In common everyday decision problems (e.g., which financial product to invest in or which drugs to buy to cure a flu) people often do not have explicit information about the full range of possible consequences and their probabilities. Instead, they can acquire sufficient information by actively exploring the structure of a decision problem to select a preferred alternative. The extent to which information about a decision problem is sampled depends on various factors (Wulff et al., 2018). For instance, research has revealed that both numerical abilities (Lejarraga, 2010; Ashby, 2017) and emotions (Frey et al., 2014) are related the exploration process measured by sample sizes in a sampling paradigm of a decision from experience task (Hertwig and Erev, 2009). However, in spite of the fact that emotions can exert a distinct effect on judgment and decision making depending on numerical abilities (Peters et al., 2006b; Peters, 2012), there is scarce research investigating this phenomenon in the context of decisions from experience. The goal of the present study is to fill this gap and to extend our understanding of how numerical abilities and emotions interact in the decision-making process. Namely, we aim to test how objective statistical numeracy and emotion of fear jointly contribute to the exploration of decision problems under uncertainty and whether the amount of acquired information predicts choices. Additionally, we examine whether the source of fear (i.e., integral vs. incidental) may influence the relationship between numeracy and search effort.

### Numeracy and Decision Making

Numerous studies have recently documented the advantage of more numerate people in making good decisions (Garcia-Retamero and Cokely, 2017; Cokely et al., 2018). For example, people who are more statistically numerate (i.e., those who better understand the concept of probability and statistical information and are able to use them efficiently; Cokely et al., 2012) are more likely to make normatively superior choices under risk (Pachur and Galesic, 2013) and are less susceptible to some biases (Reyna and Brainerd, 2008; Liberali et al., 2012), which in turn may result in their better actual health (Garcia-Retamero et al., 2015) and accumulation of wealth (Estrada-Mejia et al., 2016).

Among possible cognitive mechanisms underpinning these effects (i.e., selective allocation of attention and judgment calibration; for a detailed discussion see Garcia-Retamero and Cokely, 2017), better decisions in more numerate people are driven at least in part by the fact that such individuals deliberate more on a decision problem (i.e., they spend more time making a choice; Ghazal et al., 2014), acquire more information about potential outcomes as well as their probabilities, and employ more effortful and elaborative processing (Jasper et al., 2017), even if they rarely compute expected values to maximize payoffs (Cokely and Kelley, 2009).

**FIGURE 1:**
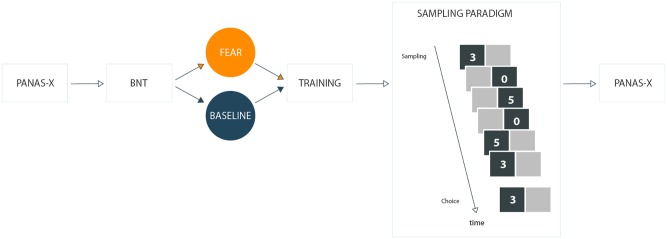
Schematic illustration of the general experimental procedure.

Properties, regarding more extensive and thorough information processing, that characterize people with high numeracy, evidently manifest themselves in making decisions under uncertainty. In laboratory settings, conditions reflecting real-life decision problems with uncertain consequences are often arranged in a decision from experience task (Wulff et al., 2018), in which participants are presented with alternatives (e.g., monetary lotteries). Without any initial knowledge concerning possible outcomes and probabilities, they can freely explore distribution of payoffs to arrive at a decision. To illustrate, in the decision from experience task participants are usually shown with two boxes representing two unknown payoff distributions (e.g., gambles A and B). Participants can freely explore these distributions by uncovering outcomes hidden under each box. For example, in a choice problem in which 3 EUR can be won with the probability of 75% and 5 EUR can be won with the probability of 25% (gamble A) or 5 EUR can be won with the probability of 30% (otherwise nothing; gamble B), participants explore two boxes representing gambles A and B. If a participant draws six samples to sequentially explore gambles A, B, B, B, A, and A, he/she may uncover possible outcomes randomly drawn from the payoff distribution (e.g., 3, 0, 5, 0, 5, and 3; see **Figure 1** for illustration). Search process is terminated, when a participant is ready to make a choice (which gamble A or B he/she prefers). The task can be fully parametrized to investigate a different number of distributions, outcomes, and different levels of probabilities.

To our best knowledge, to date at least two experiments investigated the role of numeracy in information acquisition using the decision from experience task. First, Lejarraga (2010) showed that participants with high numeracy sampled significantly more information across decision problems in comparison to people with low numeracy. Second, Ashby (2017) reported that higher numeracy was related to the larger number of samples drawn and greater consistency in choices across two choice formats (i.e., description vs. experience).

To summarize, these findings draw a picture of a highly numerate individual who deliberates more on a decision problem, exhaustively processes information and enjoys thinking about a decision problem (Lag et al., 2014; Bruine de Bruin et al., 2015). Consequently, in the decision from experience task, such individuals are likely to exhibit more effort in information search resulting in greater engagement in elaborative but also affective encoding of such content in long-term memory representation (Cokely et al., 2018).

### Emotions and Decision Making

Throughout the past three decades, increasing attention has been paid to an important role of affect, emotions and feelings in judgment and decision making (Bechara, 2000; Loewenstein and Lerner, 2003; Peters et al., 2006a; Lerner et al., 2015). The focus on emotions resulted in developing various descriptive models positing, among others, that behavior under risk can be driven by feelings (the risk-as-feelings hypothesis; Loewenstein et al., 2001), affect mediates the relationship between risks and benefits (the affect heuristic; Slovic et al., 2007) or that emotions can signal future negative consequences of choices what subsequently helps one to select an advantageous option (the somatic marker hypothesis; Bechara and Damasio, 2005).

Interestingly, it has been documented that the impact of emotions on decision making depends on appraisal tendencies—goal-directed processes that are associated with specific emotions and go beyond their valence only (Lerner and Keltner, 2000). As predicted by the Appraisal-Tendency Framework (Han et al., 2007), different emotions can trigger different cognitive predispositions to assess future events in line with appraisal dimensions that triggered these emotions. To put this in the context of information acquisition, Frey et al. (2014) found the emotion of fear (in comparison to a baseline emotional state) influenced search behavior as measured by sample size and switching frequency between alternatives. At the same time there were no credible effects of sadness and anger. Despite the same negative valence as fear, these emotions probably triggered different appraisal tendencies. The authors argue that fear could have triggered appraisals related to low certainty, high situational control and high anticipated effort which, in turn, evoked a compensatory behavioral response reflected in increased search effort—a response that could have been useful in coping with fear.

Fear is a basic emotion that signals threat in the environment (Öhman and Mineka, 2001) and prepares the survival-related response often operating without conscious experience (LeDoux, 1996). Hence, on the one hand it could serve as a cue indicating it is adaptive to collect more information about a threatening stimulus (e.g., in case of diagnosis of a severe disease, a fearful individual would search for more details regarding drugs and possible treatments). On the other hand, research concerning the impact of fear on attention indicates that task-irrelevant fearful stimuli capture attention (Vuilleumier and Schwartz, 2001; Vuilleumier et al., 2001; Fox et al., 2002), leading to impaired performance in a concurrent task and better encoding of these irrelevant stimuli in memory (Matusz et al., 2015). In case of decision tasks (the probabilistic inference task; Wichary et al., 2016), processing task-irrelevant negative and arousing stimuli (e.g., fearful stimuli) may result in attention-narrowing and focusing on the most important information which was manifested by lower search effort in the decision task (e.g., if I think a stranger on a street intends to harm me, I would not put time and effort exploring possible outcomes of selecting a better credit offer, but rather focus on the imminent danger).

We argue that these potential discrepancies regarding the effects of fear on search behavior (i.e., more vs. less information search) can be attributed to the *source* of this emotion. That is, following Lerner et al. (2015), we introduced the distinction between incidental and integral affect. While the former represents task-irrelevant affect not directly related to a decision problem and which does not guide normatively better decisions (e.g., negative mood caused by bad weather should not influence investment decisions), the latter is directly related to a decision problem and can be relevant in terms of making good choices (e.g., fear of losing money may lead to exploring various offers of savings accounts). In the present study, we expect that the source of emotion of fear (i.e., incidental and integral) would contribute to search effort in different ways, depending on numeracy.

### The Role of Emotions in Decision Making Depending on Numeracy

An interesting, however, still underexplored line of research has linked numeracy and decision making to affect (Peters et al., 2006b; Peters, 2012). For instance, Peters et al. (2006b) in a series of experiments tested a theoretical idea according to which more numerate people draw more precise affective meaning from comparison of numbers (i.e., affective precision) that in turn guides their decisions. Nevertheless, the role of affective precision in decision making is not consistent. On the one hand, the authors found that more numerate people were likely to make optimal choices–they preferred a smaller bowl with 10 jelly beans, one of which was a winning jelly bean over a larger bowl with 100 jelly beans containing nine winning jelly beans, probably because they rated feelings of goodness or badness of the former one as more clear. On the other hand, the clarity of feelings was related to suboptimal decisions in a different task. That is, people with high numeracy rated a gamble with a small loss as more attractive than a no-loss gamble and this relationship was partially mediated by affective precision.

We argue that these findings may be explained by focusing on the source of affect. For example, it has been found that people with high numeracy are less prone to incidental affect that is not directly related to a decision problem (Traczyk and Fulawka, 2016). At the same time, these individuals are more sensitive to integral affect that is elicited by a decision problem (Petrova et al., 2014).

To illustrate, in a study by Petrova et al. (2014), participants were informed that they own a camera worth 500 EUR. Depending on the experimental condition, the camera was described in a neutral or affective way, eliciting affect-poor and affect-rich contexts, respectively. Then, participants were asked to declare how much they would pay for insurance against the loss of the camera with a given probability, and to rate emotional reactions to this loss. Based on previous research (Rottenstreich and Hsee, 2001), affect-rich conditions should lead to more distorted probability weighting (reflected by the curvature of the probability weighting function) and, in consequence, lower sensitivity to changes in probability. Indeed, the study indicated that participants were less sensitive to changes in probability in the affect-rich condition and the effect was moderated by numeracy. Importantly, higher numeracy was related to higher variance in reported emotions (i.e., more numerate people reported more differentiated ratings of fear and hope to probabilities irrespective of the experimental condition) that in turn predicted higher sensitivity to probability, suggesting that more numerate participants could have extracted more affective information from probabilities that were relevant and integral to a decision problem.

Traczyk and Fulawka (2016) used a similar procedure to manipulate with irrelevant and incidental affect. In their study, participants were asked to perform two unrelated tasks: the insurance task and the perceptual task. In the insurance task, participants declared how much they would pay for insurance on a coupon worth 500 PLN (Polish Zloty), whereas in the perceptual tasks they were instructed to identify target stimuli in a stream of distractors. Depending on the experimental condition, distractors were either neutral or fearful pictures. The results showed that incidental affect led to a lower sensitivity to changes in probability, but this effect was present only in a group of less numerate participants.

Taken altogether, these results suggest that emotional states (e.g., current fear) of different sources (i.e., integral vs. incidental) can have different impacts on processing probabilities depending on the level of numeracy. It also conforms with the predictions of skilled decision theory (Cokely et al., 2018), according to which statistical numeracy directly increases the precision and calibration of affective responses resulting in affectively charged representative understanding of a decision problem. In other words, integral affect can inform more numerate people about a decision problem and motivate them to deliberate and acquire more information to make good decisions.

### Overview and Research Hypotheses

Building on the findings reported above, in the present study we expected that people with higher numeracy would put more effort in the exploration of a decision problem. Furthermore, findings regarding numeracy and affect suggest that search effort in decision problems (i.e., the amount of information acquired) should depend on the source of affect. Specifically, we hypothesized that incidental fear (i.e., a fearful state that is not related to a decision problem directly) would influence the amount of information sampled by people with low (but not high) numeracy because low numerate people are more prone to incidental affect. On the other hand, we predicted that integral fear (i.e., a fearful state elicited by a decision problem) that is meaningful to make a choice would influence search effort only in more numerate participants who are more sensitive to changes in probability and have more differentiated emotional responses to probabilities (i.e., emotional responses correlate more strongly with changes in probability). Moreover, we aimed to explore whether greater search effort would be beneficial for choices. Despite previous research investigated the interaction between numeracy and affect (incidental or integral, separately) using standard lottery tasks (e.g., decisions from description employing static lottery sets), to our best knowledge none of these works addressed the differences between integral and incidental affect and the role of numeracy in dynamic decision from experience tasks. We attempted to fill this gap.

These hypotheses were addressed in a series of two experiments.^1^ In each experiment, we employed a decision from experience task (a sampling paradigm) to investigate information search in nine binary decision problems. In Experiment 1, before the sampling task we elicited incidental fear by asking participants to recall fearful events from their life. In Experiment 2, integral fear was elicited by asking participants to make choices concerning medical treatment of a hypothetical disease they were suffering. Decision problems and their payoff distributions were the same in the two experiments and across each condition. In both experiments, we assessed objective statistical numeracy and controlled for a change in the current emotional state.

## Experiment 1

### Method

#### Participants

A total of 118 adult volunteers (69% females, *M*_age_ = 25.3, *SD* = 6.3) from the general and student populations participated in an online study for course credit or financial compensation (approximately 10 EUR). Participants received explicit information that the study investigates decision making under uncertainty. Participants provided informed consent prior to the experiment, which was approved by the Ethical Board of the SWPS University of Social Sciences and Humanities.

#### Materials and Methods

We used the following measures:

##### The positive and negative affect schedule – expanded form

The Positive and Negative Affect Schedule – Expanded Form (PANAS-X; Watson and Clark, 1999) is a comprehensive mood inventory. PANAS-X is a self-report questionnaire, confirmed to reflect the hierarchical structure of affect. It also can be used to assess both short-term and long-term individual differences in affect. The scale consists of a group of words and phrases that describe different feelings and emotions (e.g., “afraid,” “frightened,” and “cheerful”). We measured current basic negative (e.g., fear) and positive (e.g., joviality) emotional states with items drawn from the Polish adaptation of PANAS-X (Fajkowska and Marszał-Wiśniewska, 2009).

##### The Berlin Numeracy Test

The Berlin Numeracy Test (BNT; Cokely et al., 2012) is a psychometric instrument that measures risk literacy, statistical numeracy and comprehension of probability. Across numerous studies, the BNT has been shown to be an efficient research tool to measure objective numerical abilities. In the present experiment, we used the test consisting of four items presented to participants in a fixed order.

##### Decision from experience

In both experiments, we used a decision from experience task to investigate information search in nine binary problems used in previous studies (Frey et al., 2014; see **Table 1**). In each decision problem, two boxes representing two alternatives with unknown distribution of payoffs were displayed. Participants were informed that they could freely explore outcomes and frequencies by drawing random samples from each distribution. Having selected an alternative, an outcome was displayed for 1000 ms. Participants decided by themselves which alternative’s distribution they wanted to sample from, when to switch between them and when to terminate exploration. Having finished sampling, participants indicated which alternative they preferred by clicking the “Choose” button below boxes and then again on the chosen box. Prior to the experimental procedure, every participant had to pass through two training trials involving decisions from experience and provide correct responses according to instructions in this task. The purpose of this training session was to familiarize participants with procedure mechanisms (i.e., how to explore two alternatives and to choose one of them), but not to prime them with specific responses in subsequent decision problems. To achieve this goal, instead of numerical values, only geometrical shapes in different colors were selected for payoff distributions. During the training session, tips regarding procedure mechanisms were displayed on a computer screen. In the first training trial, participants were asked to explore two simultaneously presented distributions and select (choose) the one where only circles were present. A slightly different task was provided for the second training trial, where participants were asked to explore two presented distributions and select the one where a pink square was displayed more frequently. After each trial participants received feedback. If they chose a wrong distribution, they had to repeat this training trial until they responded correctly.

**Table 1:** Nine decision problems based on a study by Frey et al. (2014) that we used in the two experiments.

Decision problem	Payoff distributions		Expected values	
	
	*H*	*L*	*H*	*L*
1	4, 0.8	3, 1.0	3.2	3
2	−3, 1.0	−32, 0.1	−3	−3.2
3	−3, 1.0	−4, 0.8	−3	−3.2
4	32, 0.1	3, 1.0	3.2	3
5	32, 0.025	3, 0.25	0.8	0.75
6	3, 1.0	5, 0.55	3	2.75
7	11, 0.35	4, 0.9	3.85	3.6
8	−12, 0.25	−32, 0.1	−3	−3.2
9	−4, 0.25	−3, 0.35	−1	−1.05

*H-payoff distribution with the higher expected value (EV); L-payoff distribution with the lower EV. Values represent outcomes and their probabilities (e.g., “4, 0.8” stands for an outcome of +4 with the probability of 80%).*

#### Procedure

We randomly assigned participants to one of the two conditions (see **Figure 1**), in which we evoked either incidental fear or baseline emotion (i.e., happiness^2^). At the beginning of the experiment, we measured participants’ initial emotional state using the PANAS-X scale. Subsequently, we assessed their numeracy using the BNT. Next, participants had to complete previously described two training trials. Having finished the training session, we introduced a between-subjects manipulation with incidental emotion. That is, based on previous research that has demonstrated that mental imagery systematically evokes emotions on a declarative and physiological level (Holmes and Mathews, 2005; Traczyk et al., 2015; Sobkow et al., 2016), participants were asked to recall and write down life events in which they felt the target emotion (i.e., fearful vs. happy live events). Then, prior to the fourth and seventh decision problem, participants were asked again to recall, but this time just to imagine the previously described life events for 30 s. Finally, to track changes in emotional responses, we again measured participants’ current emotional state with the PANAS-X.

### Results

#### Manipulation Check

In order to check whether participants in the two conditions differed in fear ratings, we regressed a post-test score in the fear subscale from the PANAS-X on the experimental condition and the corresponding pretest score. We found that, controlling for pretest scores, mean post-test fear ratings were higher in the fear condition than in the baseline condition, *b* = 0.99, *p* = 0.004. Additional analyses showed that people in the fear condition were less happy in the post-test, *b* = -1.39, *p* = 0.003.

#### The Effects of Numeracy and Incidental Fear on Search Effort, Search Policy and Choice

We performed four linear regression analyses in which we predicted sample size, switching frequency, the number of choices maximizing expected value (EV) and the number of choices maximizing experienced mean returns with the same set of variables: BNT, the experimental condition and their interaction. The BNT was *z*-scored and the experimental conditions were coded as 0.5 (the fear condition) and -0.5 (the baseline condition), so the coefficients that include the effect of the experimental condition (i.e., the main effect and interaction terms) can be directly interpreted as a difference between fear and baseline. Despite skewed distributions of dependent variables, we report results using untransformed data for a more straightforward interpretation of regression coefficients (but our main findings also hold when square root transformation or Poisson regression were applied). The relationships among variables used in Experiment 1 are presented in **Table 2**. There were five people who correctly answered four items of the BNT. Ten participants answered three items; 27 participants answered two items; 30 participants answered one item. Forty three people did not answer any item correctly.

**Table 2:** The relationships among measures used in Experiment 1.

	1	2	3	4	5	6
1. BNT	–					
2. Mean sample size	0.304***	–				
3. Switching rate	−0.175	−0.427***	–			
4. EV choices	0.013	−0.002	−0.041	–		
5. Experienced mean returns	0.317***	0.34***	0.179	−0.044	–	
6. Change in fear ratings	0.096	0.004	0.013	0.012	−0.101	–

*The change in fear ratings was computed by subtracting pretest fear ratings from post-test fear ratings as measured with PANAS-X. ^∗^p < 0.05, ^∗∗^p < 0.01, ^∗∗∗^p < 0.001.*

##### Search effort

**FIGURE 2:**
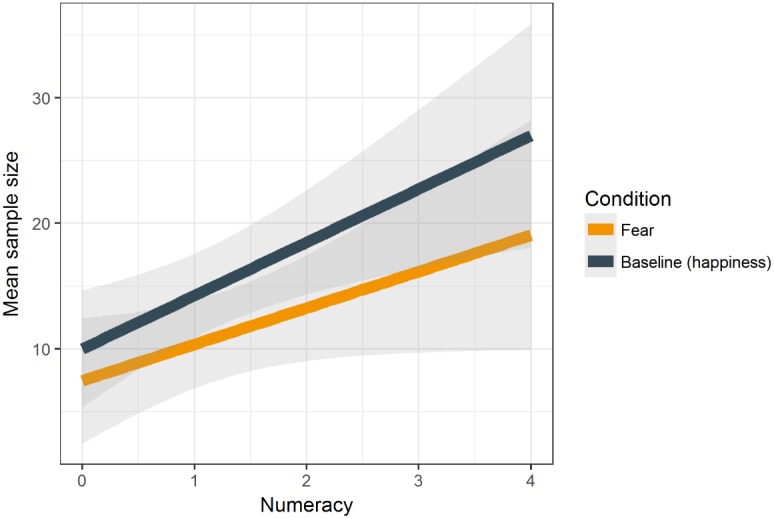
Mean sample size as a function of numeracy and the experimental condition (incidental fear vs. baseline condition – happiness).

We averaged sample sizes across nine decision problems per each participant^3^ (*M* = 12.93, *SD* = 13.50). That is, we summed up samples per each decision problem for each participant. Then, the arithmetic mean of these samples was calculated for each participant. Next, we regressed mean sample size on numeracy, the experimental condition and their interaction. As predicted, numeracy was positively related to the number of samples drawn, *b* = 4.13, *p* < 0.001 (**Figure 2**). Furthermore, we observed a marginally significant effect of incidental fear on the sample size, *b* = -4.12, *p* = 0.089.^4^ Individuals sampled more in the baseline condition in comparison to the fear condition. We did not find an interaction effect of numeracy and the experimental condition on sample size, *b* = -1.56, *p* = 0.520 (see **Table 3** for details).

**Table 3:** Results of regression analyses predicting sample size, switching frequency and choices maximizing EV in Experiment 1.

Predictor	Sample size			Switching frequency			EV choices			Experienced mean returns		
	
	*b*	95% CI	*p*	*b*	95% CI	*p*	*b*	95% CI	*p*	*b*	95% CI	*p*
Intercept	12.90^∗∗^	(10.52, 15.27)	<0.001	0.49^∗∗^	(0.42, 0.56)	<0.001	3.99^∗∗^	(3.76, 4.22)	<0.001	5.49	(5.1, 5.83)	<0.001
Numeracy	4.13^∗∗^	(1.74, 6.51)	<0.001	−0.06^†^	(−0.13, 0.01)	0.071	0.01	(−0.23, 0.24)	0.954	0.66^∗∗∗^	(0.31, 1.01)	<0.001
Group (Fear)	−4.12^†^	(−8.86, 0.64)	0.089	−0.04	(−0.18, 0.10)	0.632	0.28	(−0.18, 0.74)	0.238	−0.92^∗^	(−1.6,−0.22)	0.010
Numeracy^∗^Group	−1.56	(−6.32, 3.22)	0.520	0.1	(−0.04, 0.24)	0.134	−0.32	(−0.78, 0.16)	0.186	0.46	(−0.24, 1.16)	0.190
	*R*^2^ = 0.119^∗∗^			*R*^2^ = 0.052			*R*^2^ = 0.028			*R*^2^ = 0.165^∗∗∗^		

*Group was coded as 0.5 (Fear) and -0.5 (Baseline). ^†^p < 0.1, ^∗^p < 0.05, ^∗∗^p < 0.01, ^∗∗∗^p < 0.001.*

**Table 4:** Results of regression analyses predicting experienced mean returns in Experiment 1 and Experiment 2.

Predictor	Experiment 1			Experiment 2		
	
	*b*	95% CI	*p*	*b*	95% CI	*p*
Intercept	5.53	(5.16, 5.90)	<0.001	5.71	(5.19, 6.23)	<0.001
Numeracy	0.50^∗^	(0.11, 0.89)	0.012	0.06	(−0.52, 0.63)	0.841
Group (Fear)	−0.74^†^	(−1.48, 0.00)	0.051	−0.38	(−1.40, 0.66)	0.470
Sample size	0.51^∗∗^	(0.13, 0.89)	0.008	1.22^∗∗^	(0.40, 2.04)	0.004
Numeracy^∗^Group	0.42	(−0.36, 1.18)	0.296	0.42	(−0.72, 1.56)	0.470
Numeracy^∗^Sample size	−0.11	(−0.54, 0.33)	0.628	−0.25	(−1.22, 0.71)	0.603
Group^∗^Sample size	0.16	(−0.58, 0.92)	0.661	−0.84	(−2.48, 0.78)	0.307
Numeracy^∗^Group^∗^Sample size	−0.10	(−0.96, 0.78)	0.827	−0.68	(−2.62, 1.24)	0.480
	*R*^2^ = 0.220^∗∗∗^			*R*^2^ = 0.134^∗∗∗^		

*Group was coded as 0.5 (Fear) and -0.5 (Baseline). ^†^p < 0.1, ^∗^p < 0.05, ^∗∗^p < 0.01, ^∗∗∗^p < 0.001.*

##### Search policy

We analyzed switching behavior following the same procedure as Hills and Hertwig (2010). That is, for each individual and a decision problem we calculated the ratio between the number of actual switches between alternatives and the maximum number of possible switches (i.e., *n*–1, with *n* being the total number of drawn samples). Average switching frequency was *M* = 0.49 (*SD* = 0.37). Consistent with other findings (Hills and Hertwig, 2012), switching frequency was negatively correlated with sample size, *r =* -0.43, *p* < 0.001.

In the regression analysis, we found that BNT was marginally related to switching frequency, *b* = -0.63, *p* = 0.071, suggesting that more numerate participants were switching between two alternatives less frequently. The experimental condition and the interaction between numeracy and the experimental condition did not significantly predict the number of switches between alternatives (*p*s > 0.05).

##### Expected value maximization

We summed up a total number of choices consistent with the EV maximization principle (*M* = 3.98, *SD* = 1.26) to test the effects of numeracy, the experimental condition and their interaction on this measure. None of these predictors significantly explained the number of choices with higher EV (all *p*s > 0.05).

##### Experienced mean returns maximization

Hertwig and Pleskac (2008) assumed that people derive their choices from differences in the samples’ mean return. The maximization of experienced mean returns predicted more choices in the decision from experience format than expected value in the description format (Wulff et al., 2018). Therefore, we calculated the experienced mean returns summing all the experienced outcomes in the respective decks and dividing them by respective sample sizes. The average number of choices with higher experienced mean returns was *M* = 5.5 (*SD* = 2.02). We found that more numerate individuals tended to choose options with higher experienced mean returns, *b* = 0.66, *p* < 0.001. Interestingly, we observed the main effect of the experimental condition, *b* = -0.92, *p* = 0.010. Participants in the fear condition made fewer choices consistent with the experienced mean returns maximization than those in the baseline condition. We did not find an interaction effect of numeracy and group (see **Table 3**).

We further explored these effects by introducing mean sample size to the model (**Table 4**). The effects of BNT and fear did hold. Additionally, we found that the number of choices maximizing experienced mean returns was predicted by mean sample size, *b* = 0.51, *p* = 0.008.

### Summary

To summarize, results of Experiment 1 supported our main hypothesis, according to which more numerate participants will exhibit more search effort manifested in larger sample sizes. However, opposite to our predictions, incidental fear did not influence search effort in case of people with low numeracy. That is, despite a weak main effect of incidental fear on search effort, numeracy did not moderate this relationship. Moreover, we observed that numeracy, incidental fear and search effort (as measured by mean sample size) predicted the number of choices maximizing experienced mean return. It suggests that more numerate people sampled more information about a decision problem and, in turn, chose more advantageous alternatives based on experienced outcomes.

In Experiment 2, we introduced a procedure modification to test these relationships for a different source of fear: integral fear.

## Experiment 2

### Method

#### Participants

Ninety adult volunteers (60% females, *M*_age_ = 26.4, *SD* = 6.7) took part in Experiment 2. Participants were recruited from the same population as in Experiment 1; they were incentivized in the same way and received the same initial information about the study. The experiment was approved by the Ethical Board of the SWPS University of Social Sciences and Humanities.

#### Materials and Methods

We used the same materials and method as in Experiment 1.

#### Procedure

To elicit integral fear, we randomly assigned participants to one of the two between-subjects conditions: medical decisions or financial decisions. In general, we expected the former condition to be relatively more frightening than the latter one because of affect-rich medical outcomes (Pachur et al., 2014; Suter et al., 2015). In Experiment 2, we used almost the same procedure as in Experiment 1. At the outset, we measured initial participants’ current emotional state with the PANAS-X. Then we measured numeracy with the BNT, followed by the previously described training trials and decision problems.

The only difference in Experiment 2 was a method of inducing a desirable emotional state. Instead of incidental fear, we used integral fear manipulation. In the medical condition, participants were asked to imagine they were diagnosed with a dangerous, lethal disease. During the experimental procedure, they had to choose one of two drugs (choice alternatives), which could accelerate or slow down the development of the disease, based on an underlying distribution of payoffs. In other words, a distribution of payoffs in the decision task indicated how many years a treatment with a particular drug could prolong or shorten life given that a disease was diagnosed (e.g., an outcome of “3” informed participants that a patient randomly drawn from a population of patients who underwent a treatment with a particular drug lived 3 years longer than expected; see **Supplementary Table S1** for the exact instructions). In the financial condition, participants were asked to imagine they are CEOs of a new company and their task is to choose between two financial products, which could be beneficial or disadvantageous investments from the perspective of the company. As in the medical condition, potential outcomes of financial products were hidden under two boxes representing payoff distributions of the two alternative investments. In both conditions, participants faced nine decision problems with the same payoff distributions as in Experiment 1. At the end, we once again measured their current emotional state with the PANAS-X.

### Results

All analyses reported below followed the approach we employed in Experiment 1. One participant did not complete the whole procedure and was excluded from further analyses.^5^ The relationships among variables used in Experiment 2 are presented in **Table 5**. There were nine people who correctly answered four items of the BNT. Nine participants answered three items; 22 participants answered two questions; 23 participants answered one item. Twenty five people did not answer any item correctly.

**Table 5:** The relationships among measures used in Experiment 2.

	1	2	3	4	5	6
1. BNT	–					
2. Mean sample size	0.328**	–				
3. Switching rate	−0.286**	−0.44***	–			
4. EV choices	0.099	0.095	−0.029	–		
5. Experienced mean returns	0.08	0.163	0.059	0.009	–	
6. Change in fear ratings	0.142	−0.067	−0.103	0.075	−0.041	–

*Change in fear ratings was computed by subtracting pretest fear ratings from post-test fear ratings as measured with PANAS-X. ^∗^p < 0.05, ^∗∗^p < 0.01, ^∗∗∗^p < 0.001.*

#### Manipulation Check

To check the effectiveness of the experimental manipulation, we regressed a post-test score in the fear scale from PANAS-X on the experimental conditions (i.e., medical vs. financial outcomes) and the corresponding pretest score. Controlling for pretest scores, mean post-test fear ratings were higher in the medical condition than in the financial condition, *b* = 1.86, *p* = 0.019, which supports our prediction that medical outcomes would evoke more fearful responses in comparison to financial outcomes. We did not observe significant differences in the level of happiness between the medical condition and the financial condition, *b* = -0.32, *p* = 0.652.

#### The Effects of Numeracy and Integral Fear on Search Effort, Search Policy and Choice

##### Search effort

**FIGURE 3:**
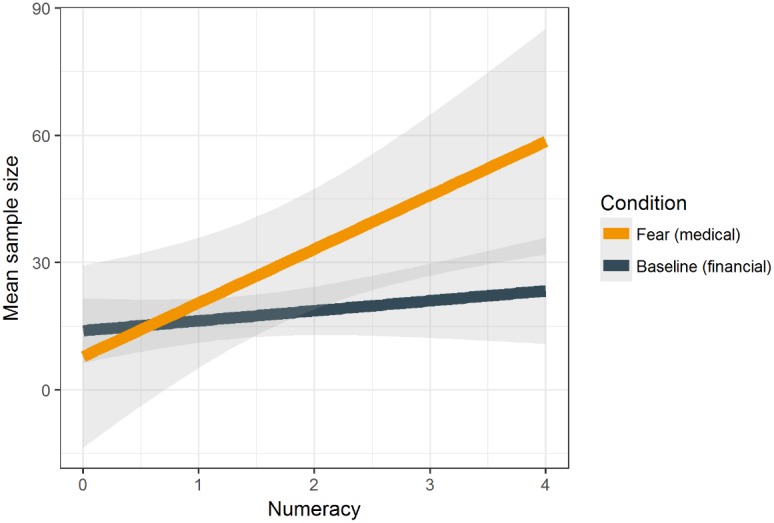
Mean sample size as a function of numeracy and the experimental condition (integral fear – medical problems vs. baseline condition – financial problems).

We averaged sample sizes across nine decision problems from each participant (*M* = 22.14, *SD* = 32.36). Replicating our previous findings, we found that the BNT was positively related to sample size, *b* = 9.67, *p* = 0.003. There was no main effect of the experimental condition, *b* = 9.16, *p* = 0.165. However, we found a significant interaction of these predictors, *b* = 13.28, *p* = 0.044 (**Figure 3**). As we expected, a simple effects analysis performed for each level of numeracy confirmed that participants with higher numeracy (those who gave correct responses in three or four items in the BNT) explored payoff distributions to a greater extent in the integral fear medical condition in comparison to the baseline financial condition (*p* = 0.043 and *p* = 0.030 for people correctly answering three and four items in the BNT, respectively).

Additionally, we tested whether sample sizes differed between Study 1 and Study 2. We found that in Study 2 participants drew significantly more samples (22 samples) than in Study 1 (13 samples), *t*(110.23) = -2.5, *p* = 0.013. This suggests that integral affect (Study 2), in comparison to incidental affect (Study 1), might have increased search effort.

##### Search policy

Average switching frequency was *M* = 0.41 (*SD* = 0.39). Results of regression indicated that participants with higher numeracy alternated back and forth between lotteries less frequently, *b* = -0.11, *p* = 0.011. Nonetheless, the experimental condition and its interaction with BNT were not significant predictors of switching frequency (*p*s > 0.5). Moreover, we corroborated results from Experiment 1, showing that a correlation between total sample size and switching frequency was negative, *r* = -0.44, *p* < 0.001.

##### Expected value maximization

On average, participants selected *M* = 4.49 (*SD* = 1.59) alternatives with higher EV. As with Experiment 1, no predictors were significantly related to the number of decisions consistent with EV maximization principle (all *p*s > 0.05).

##### Experienced mean returns maximization

We calculated experienced mean returns following the same rationale as in the previous experiment. Average number of choices with higher experienced mean returns was *M* = 5.53 (*SD* = 2.28). We did not replicate findings from Experiment 1. None of the predictors substantially explained variance of the experienced mean returns (see **Table 6**).

**Table 6:** Results of regression analyses predicting sample size, switching frequency, choices maximizing EV and experienced mean returns in Experiment 2.

	Sample size			Switching frequency			EV choices			Experienced mean returns		
	
Predictor	*b*	95% CI	*p*	*b*	95% CI	*p*	*b*	95% CI	*p*	*b*	95% CI	*p*
Intercept	22.01^∗∗^	(15.51, 28.52)	<0.001	0.40^∗∗^	(0.32, 0.48)	<0.001	4.46^∗∗^	(4.11, 4.81)	<0.001	5.47	(4.98, 5.97)	<0.001
**Numeracy**	9.67^∗∗^	(3.20, 16.13)	0.003	−0.11^∗^	(−0.19,−0.03)	0.011	0.17	(−0.17, 0.52)	0.325	0.2	(−0.3, 0.69)	0.430
Group (Fear)	9.16	(−3.84, 22.18)	0.165	−0.08	(−0.24, 0.08)	0.363	−0.28	(−0.98, 0.40)	0.417	−0.38	(−1.38, 0.62)	0.453
**Numeracy^∗^Group**	13.28^∗^	(0.36, 26.22)	0.044	0.00	(−0.16, 0.18)	0.915	0.12	(−0.58, 0.80)	0.742	0.44	(−0.54, 1.44)	0.373
	*R*^2^ = 0.169^∗∗^			*R*^2^ = 0.091			*R*^2^ = 0.019			*R*^2^ = 0.022		

*Group was coded as 0.5 (Fear) and -0.5 (Baseline). ^∗^p < 0.05, ^∗∗^p < 0.01, ^∗∗∗^p < 0.001.*

When mean sample size was introduced to the model, we again found that choices maximizing experienced mean returns significantly were predicted by mean sample size, *b* = 1.22, *p* = 0.004 (**Table 4**).

### Summary

We found that numeracy was the strongest predictor of search effort. Interestingly, although integral fear evoked by medical problems did not influence search effort directly, this relationship was moderated by numeracy. In particular, participants with higher numeracy tended to draw more samples in medical decision problems that elicited a greater integral fear. However, none of the predictors were related to choices maximizing EV. Nevertheless, sample size, but not numeracy or the experimental condition, predicted the number of choices maximizing experienced mean returns. Therefore, the relationship between numeracy and choice was not as straightforward as in Experiment 1. The pattern of results suggests that in the case of the integral fear manipulation, numeracy may influence choices indirectly by increasing search effort.

## General Discussion

The goal of the present study was to investigate the role of numeracy and emotions in decision-making process. Specifically, we used a decision from experience task to measure search effort as a function of statistical numeracy and fear. In two experiments, we found that numeracy is a robust predictor of search effort. In particular, more numerate participants tended to acquire more information about outcomes and their probabilities. Interestingly, numeracy moderated the relationship between fear and search effort but only when the source of this emotion was integral to a decision problem. That is, when fear was produced by outcomes and probabilities of the decision problem rather than being elicited by a concurrent and unrelated task, more numerate people draw more samples in comparison to people with low numeracy. These findings imply that people with high numeracy may use relevant affect as an additional cue when processing information about a decision problem. Additionally, we demonstrated that numeracy and fear did not increase the number of choices that maximized EV, but they predicted choices maximizing the experienced mean returns. Nevertheless, the nature of this relationship is not straightforward. That is, numeracy was directly related to choices in Experiment 1, while in Experiment 2, numeracy may have operated indirectly through increased search effort.

### The Adaptive Role of Fear and Numeracy in Information Acquisition

Fear, as one of the basic emotions present among humans and other primates (Ekman, 1992), informs an organism about dangers and prepares responses to a potential environmental threat (Öhman and Mineka, 2001). In this sense fear plays an adaptive and survival-related function, as it focuses attention on a threatening stimulus and motivates an organism to cope with the threat or avoid it (Adolphs, 2013). A threatening stimulus can trigger a pattern of conditioned and unconditioned behavioral responses to danger or activate “survival circuits” responsible for producing an adaptive defense response to such stimuli (LeDoux, 2014). However, the automatic survival-related response to fear may become maladaptive if it holds attentional resources and prevents an individual from processing a more important concurrent task not directly related to a threatening stimulus.

In case of decision-making research, reported effects of negative affect (e.g., fear) on process-tracing measures are mixed. For example, emotional stress (evoked by task-irrelevant high-arousal pictures of negative valence) reduced information search and promoted simplified processing (Wichary et al., 2016). Similarly, choices in the affect-rich medical domain (i.e., the choice between two medications with negative side-effects of different severity) were associated with lower sample sizes in comparison to the monetary domain (Lejarraga et al., 2016). On the other hand, fearful participants put more effort in the exploration of payoff distributions (Frey et al., 2014) in comparison to the baseline happy condition. This supports the notion that the negative affect is associated with more in-depth and elaborate information processing (for a theoretical account see Forgas, 1995; Schwarz, 2001).

Conclusions from our study suggest there are at least two important factors that offer an opportunity to explain inconsistent findings regarding the role of fear (and more generally emotions) in searching information about a decision problem. These factors are the source of fear and numeracy.

The adaptive and informational advantage of fear is contingent on whether the fear is relevant or irrelevant to a decision problem. Such distinctions have already been noticed in communication and persuasion studies showing that a negative emotional state (e.g., fear) increased motivation to elaborate when the threat was relevant and integral to a stimulus, but decreased motivation to elaborate on information that is incidental and irrelevant (Baron et al., 1994). The distinction between incidental and integral affect/emotions has also been raised in judgment and decision-making research (e.g., Lerner et al., 2015; Västfjäll et al., 2016). In line with these theoretical considerations, our study demonstrated that a state of fear elicited incidentally by an unrelated task led to lower search effort. This effect could have been driven by fearful mental images that captured and absorbed people’s attention, drawing it away from the exploration of choice alternatives. Moreover, fear serves as a cue in goal queuing (Simon, 1967): It may interrupt the current program and give high priority to real-time needs. In this case, people could have been more motivated to cope with fearful mental images instead of performing experimental tasks.

On the contrary, the state of fear elicited by the properties of a decision task could have focused people’s attention and motivation on the decision problem, increasing search effort and decreasing the switching rate. Interestingly, integral fear had an impact on search effort only among more numerate participants who are more sensitive to number-related affective reactions (Peters, 2012; Petrova et al., 2014; Peters and Bjalkebring, 2015) and are able to derive a richer gist of information (Reyna, 2004; Reyna et al., 2009). Consequently, integral fear can be adaptive in such problems because it gives more priority to the experimental task and influences search effort as well as a more extensive exploration of important features of a decision problem. Since we did not test attentional engagement in these tasks, future studies using eye tracking or psychophysiological methods could address this hypothesis directly.

### The Prediction of Choices

It has recently been theorized (Cokely et al., 2018), as well as empirically confirmed (Jasper et al., 2013; Traczyk et al., 2018) that people with high numeracy exhibit more adaptive behavior under risk and uncertainty. At the level of information processing, we can conclude that more numerate people in our study were adaptive: They put in more effort when fear was integral to the problem, yet were not influenced by incidental fear. Under such conditions using integral fear as information is adaptive, as it allows one to acquire more information about the important problem before making a final decision. At the level of choice, the question of adaptive behavior is more complicated because of the variety of criteria defining good choices (Hogarth, 2015).

In our study, higher numeracy did not predict choices maximizing EV. This is not surprising in light of other research that showed the lack of such a relationship (Ashby, 2017) or a moderating role of other factors in the relationship between numeracy and choices maximizing EV (Traczyk et al., 2018). Moreover, in contrast to previous research (Frey et al., 2014), we did not observe the effect of fear on maximizing EV. These results can in part be explained by the fact that people who took part in our experiments were not paid contingent on their actual choices. Another explanation is that more numerate people followed different criteria of good choices instead of normative standards (e.g., maximizing expected value or expected utility). Accordingly, they could have drawn “enough” samples to make a satisfactory choice that maximized returns based on experienced outcomes. Additionally, it has been demonstrated that more numerate people often do not compute EV of a gamble but rather employ elaborative heuristic processing (Cokely and Kelley, 2009). That is, people with high numeracy consider more aspects of a choice problem, they recode probabilities, focus on maximum and minimum differences between outcomes or take their risk preference into account. This may result in longer deliberation about problem leading to superior choices.

Across two experiments, numeracy predicted search effort. This was positively related to more choices maximizing the experienced mean returns. It suggests that search effort may be a key factor explaining good choices. Nevertheless, the relationship between these measures is not straightforward. In Experiment 1 numeracy predicted choices maximizing experienced mean returns directly irrespective of incidental fear, while in Experiment 2 participants with high numeracy were likely to sample more information, which successively influenced choices.

An interesting question that emerges from our findings addresses the role of numeracy in search/exploratory behavior in general. In the manuscript, we reported that higher sampling was related to lower switching rate (people who generally sampled more also switched less between alternatives). In case of numeracy, Ashby (2017) showed that higher numeracy was related to lower switching rate. Our additional analyses corroborated this result. It may imply that more numerate people extensively explored only one of the two options. However, it is more plausible that people (particularly more numerate individuals) used rather a comprehensive strategy of sampling than a piecewise strategy. The piecewise search oscillates between options, each time drawing the smallest possible sample. On the other hand, a decision-maker who applies the comprehensive policy samples extensively from one option and then samples extensively from the other option. Therefore, highly numerate people could less frequently alternate back and forth between options, but, at the same time, it does not necessarily mean that they tended to sample more from one option while ignoring the other, because this alternating could be equal across all options.

We believe these findings may contribute to better understanding of the role of numeracy and fear in decision making, but also may have some practical applications. Further studies may precisely tackle this issue. For example, it seems appealing to investigate methods of increasing search effort in less numerate people by directing their attention to integral, fear-related aspects of a decision problem. Such interventions could support less numerate people in sampling more information resulting in better choices. The emotion-based intervention might be a complementary to other aids (e.g., visual aids) designed to improve risk literacy.

### Limitations and Future Research

Although we tried to minimize differences in experimental procedures between Study 1 and Study 2 in order to compare the effects of incidental and integral affect, there are still some concerns regarding comparability of our tasks. That is, keeping the same payoff distributions and choice problems, the procedures differed in instructions provided to participants. In Study 1 participants were instructed to imagine events from their life while in Study 2, we only manipulated the content of instructions (e.g., financial vs. medical scenarios). As a result, we found that integral but not incidental affect influenced people with high numeracy who searched for more information. Additionally, we demonstrated that in general, integral affect was related to more search effort in comparison to incidental affect. This suggests that integral affect is likely to increase search effort. However, an alternative explanation is also plausible. The differences in procedures might have influenced motivation or engagement that led to more extensive search. Moreover, one could argue that the experimental manipulation in Study 2 was not a direct manipulation of integral fear and it could have also influenced (or activated) other constructs that may be potentially related to motivation (e.g., mortality salience in the medical condition or money priming in the financial condition, Zaleskiewicz et al., 2013). This issue could be addressed by using other methods to compare medical and financial gambles (Lejarraga et al., 2016) and to control for potentially confounding variables.

Another interesting line of future research is to investigate whether more numerate people are more sensitive to integral emotions or rather they are able to experience integral emotions more accurately because of their previous personal experience. For instance, if a person had more personal experience with financial decisions that resulted in losses than with medical decisions, he/she may be more sensitive to such affective influences or experience them with more intensity. Furthermore, numeracy may moderate this relationship.

Finally, because numeracy has not been experimentally manipulated in our study (for an example of a study with manipulation intended to improve numeracy see Peters et al., 2017), drawing causal inferences about the influence of numeracy on decision making seems problematic. Numeracy is correlated with many measures such as intelligence (Lag et al., 2014), need for cognition (Bruine de Bruin et al., 2015), cognitive reflection (Weller et al., 2013) and education (Ghazal et al., 2014), so it cannot be excluded that one of these factors was responsible for the effects obtained in the current study. Nevertheless, previous research has demonstrated that the effects of numeracy hold even when controlling for the above mentioned variables, suggesting that numeracy is a robust unique predictor of superior decision making (Cokely et al., 2018).

### Conclusion

To summarize, our study demonstrated that people with high numeracy acquire more information about a decision problem. Importantly, more numerate people seem to use task-relevant affective information as a cue signaling the importance of a decision problem. This in turn motivates them to put more effort in the exploration of outcomes and their probabilities. In consequence of greater search effort, people with high numeracy are able to maximize experienced mean return. Altogether, decisions made by highly numerate people may be guided not only by objective properties of choice problems (e.g., outcomes), but also by adaptive affective responses to these problems.

## Data Availability Statement

All analyzed datasets for this study are included in the manuscript and the **supplementary files**.

## Author Contributions

JT developed the study concept and acquired funding. JT, AS, DL, JS, PT, KF, AP, KS, and PZ contributed equally to the study design. DL, JS, and JT programmed the procedure. DL and JT performed the data analysis. JT, AS, DL, JS, PT, KF, AP, KS, and PZ conducted the study, wrote and reviewed the article.

## Conflict of Interest Statement

The authors declare that the research was conducted in the absence of any commercial or financial relationships that could be construed as a potential conflict of interest.

## Footnotes

1 In addition to our main hypotheses regarding search effort, we also measured switching frequency. Following Frey et al. (2014) we use terms *search effort* and *switching frequency* to refer to mean sample size and mean switching frequency which together can describe search/exploratory behavior. However, effects of numeracy and fear on the latter measure were not our main interest in this study.

2 The method of inducing incidental affect was inspired by the study by Frey et al. (2014) who manipulated with different emotional states (i.e., fear, sadness, and anger) in comparison to a baseline emotional state of happiness.

3 Three participants were excluded from all analyses in Experiment 1 because they did not draw any sample in any of the problems in the entire experiment.

4 When we applied a square root transformation to dependent variable, the effect became significant, *b* = -0.62, *p* = 0.045. Performing Poisson regression with number of samples drawn from the whole experiment as the criterion variable (which is often employed to count data with a right-skewed distribution) led to similar conclusions, *b* = -0.34, *p* < 0.001.

5 The overall pattern of results did not change when we included data from this participant.

## Supplementary Material

DATA SHEET S1 Data set - Experiment 1.

DATA SHEET S2 Data set - Experiment 2.

DATA SHEET S3 Description of data sets and variables.

TABLE S1 Instructions presented to participants in two experiments.
