# Supplementary material for: Does Fear Increase Search Effort in More Numerate People? An Experimental Study Investigating Information Acquisition in a Decision From Experience Task
Source: Front Psychol. 2018 Aug 3;9:1203. doi: 10.3389/fpsyg.2018.01203 (PMC6085433; doi:10.3389/fpsyg.2018.01203)
Supplement: DATA SHEET S3 — Description of data sets and variables. [file Data_Sheet_3.DOCX]

Description of data sets and variables

Files:

dfe1_perpers.csv - DFE data aggregated per person from Experiment 1 (incidental affect)

dfe2_perpers.csv - DFE data aggregated per person from Experiment 2 (integral affect)

Names of variables are the same for the dfe1_perpers.txt and dfe2_perpers.txt.

Variables:

samples_mean - Mean number of samples

samples_exp - Number of samples drawn from the whole experiment

samples_sqrt - Square root transformation of the samples_mean variable

swrate - Mean switching rate

EV - Number of choices consistent with EV maximization principle

max_return - Number of choices consistent with maximization of the experienced mean returns

BNT - Berlin Numeracy Test score

BNT_z – Berlin Numeracy Test z-scores

condition – dummy coded condition:

- Study 1 – incidental affect (1 – fear, -1 – baseline)
- Study 2 – integral affect (1 – medical, -1 – financial)

pret_F – scores from the PANAS-X fear scale in the pre-test

post_F - scores from the PANAS-X fear scale in the post-test

pret_J – scores from the PANAS-X joviality scale in the pre-test

post_J - scores from the PANAS-X joviality scale in the post-test
