# Supplementary material for: Does Fear Increase Search Effort in More Numerate People? An Experimental Study Investigating Information Acquisition in a Decision From Experience Task
Source: Front Psychol. 2018 Aug 3;9:1203. doi: 10.3389/fpsyg.2018.01203 (PMC6085433; doi:10.3389/fpsyg.2018.01203)
Supplement: TABLE S1 — Instructions presented to participants in two experiments. [file Table_1.DOCX]

Supplementary Material

Does fear increase search effort in more numerate people? An experimental study investigating information acquisition in a decision from experience task

**Jakub Traczyk*, Dominik Lenda, Jakub Serek, Kamil Fulawka, Pawel Tomczak, Karol Strizyk, Anna Polec, Piotr Zjawiony, Agata Sobkow**

*** Correspondence:** Jakub Traczyk: [jtraczyk@swps.edu.pl](mailto:jtraczyk@swps.edu.pl)

# Table S1. Instructions presented to participants in two experiments

## Experiment 1

### Instructions

In this task, you will be asked to make decisions regarding nine decision problems. Each decision problem contains two monetary lotteries. Your task is to indicate which lottery from a given pair seems more attractive to you – in other words, your task is to choose the lottery that you would like to play out as if real money were involved.

On the left- and right-hand side of the screen you will see two lotteries. The possible outcomes of those results are hidden behind gray boxes. You may uncover the values hidden behind those boxes – those values represent outcomes that could be obtained if the lottery was actually played out (it could be either a loss, gain or zero).

Outcomes hidden behind the gray boxes appear with a given frequency, which represents an actual chance of getting this outcome (each decision problem contains maximally four different outcomes).

You can uncover outcomes hidden behind the boxes as many times as you want, until you are ready to choose the lottery that is more appealing to you. If you don’t want to uncover any more potential outcomes, use the left-mouse button to click the “Choose” button.

If you want to uncover an outcome of the lottery on the left, use the left-mouse button to click the box on the left.

If you want to uncover an outcome of the lottery on the right, use the right-mouse button to click the box on the right.

If you are ready to choose the lottery that you prefer, use the left-mouse button to click the “Choose” button … and then pick the lottery that seems more attractive to you by clicking the left or right box.

Take as much time as you need to complete the tasks. Press the ENTER button to start the study.

### Reminder

If you want to uncover an outcome on the left side, use the left-mouse button to click the box on the left.

If you want to uncover an outcome on the right side, use the left-mouse button to click the box on the right.

If you are ready to make a decision, use the left-mouse button to click the “Choose” button and then pick the lottery that seems more attractive to you by clicking the left or right box.

Take as much time as you need to complete the tasks.

Press the “Forward” button using the mouse to start the study

This is the end of this part of the study.

On the next page, you will be asked to fill out the last questionnaire.

Thank you for your help!

### Manipulation

#### Incidental fear condition

Bring back memories of events from your life that led to a feeling of FEAR, visualize them in your head and describe briefly (1-2 sentences).

Information provided in this study is fully anonymous and will be automatically deleted after completing the research; however, you will be asked to conjure up those memories several times during the tasks.

#### Baseline condition (happiness)

Bring back memories of events from your life that led to a feeling of HAPPINESS, visualize them in your head and describe briefly (1-2 sentences).

Information provided in this study is fully anonymous and will be automatically deleted after completing the research; however, you will be asked to conjure up those memories several times during the tasks.

## Experiment 2

### Integral fear condition (medical decisions)

Imagine that you were diagnosed with a severe, fatal disease. Your task is to make several decisions regarding nine medical problems, which are crucial to the development of the disease during its early stages.

In each problem you will have to make a choice between two drugs, which may accelerate or slow down development of the disease. Results of using those drugs are uncertain, but you may check what were the effects of those drugs relative to other people who were diagnosed with the same disease.

Possible outcomes of the drugs are hidden behind two gray boxes, which are placed on the left- and right-hand side of the screen. You may uncover the values hidden behind the boxes to see what kind of an outcome to expect from a given drug.

For example, if you uncover the left box 10 times which resulted in seeing value +2 for 5 times and value -1 for 5 times, it means that for every 10 people that chose this drug 5 of them lived 2 years longer and 5 of them lived 1 year shorter, in comparison to people who did not take any drug at all.

When uncovering possible outcomes, values appear with a given frequency that represents an actual chance of getting this outcome (each decision problem contains maximally four different outcomes).

You can uncover outcomes hidden behind the boxes as many times as you want, until you are ready to choose the drug that is more appealing to you. If you don’t want to uncover any more potential outcomes, use left-mouse button to click the “Choose” button.

If you want to uncover a possible outcome of using the drug on the left, use the left-mouse button to click the box on the left.

If you want to uncover a possible outcome of using the drug on the right, use the left-mouse button to click the box on the right.

If you are ready to choose the drug that you prefer, use the left-mouse button to click the “Choose” button and then pick the drug that seems more attractive to you by clicking the left or right box.

Take as much time as you need to complete the tasks.

Press the “Next” button using the mouse to start the study.

### Baseline condition (financial decisions)

Imagine that you are running your newly started company. Your task is to make several decisions regarding nine financial problems, which are crucial to the development of your company during the early stages.

In each problem, you will have to make a choice between two financial products, which may accelerate or slow down company growth. Investment outcomes in those products are uncertain, but you may check what were the financial results of those decisions in cases of other business owners.

Possible outcomes of the investments are hidden behind two gray boxes, which are placed on the left- and right-hand side of the screen. You may uncover the outcomes hidden behind the boxes in to see what kind of an outcome to expect from a given financial product.

For example, if you uncover the left box 10 times which resulted in seeing value +2 for 5 times and value -1 for 5 times, it means that for every 10 people that chose this financial product 5 of them gained 2PLN and 5 of them lost 1PLN in comparison to people who did not invest at all.

When uncovering possible outcomes, outcomes appear with a given frequency that represents an actual chance of getting this outcome (each decision problem contains maximally four different outcomes).

You can uncover values hidden behind the boxes as many times as you want, until you are ready to choose the lottery that is more appealing to you. If you don’t want to uncover any more potential outcomes, use the left-mouse button to click the “Choose” button.

If you want to uncover an outcome of the product on the left, use the left-mouse button to click the box on the left.

If you want to uncover an outcome of the product on the right, use the left-mouse button to click the box on the right.

If you are ready to choose the product that you prefer, use the left-mouse button to click the “Choose” button and then pick the product that seems more attractive to you by clicking the left or right box.

Take as much time as you need to complete the tasks.

Press the ’Next’ button using the mouse to start the study
